# Immobilization of Ferrocene-Modified SNAP-Fusion Proteins

**DOI:** 10.3390/ijms14024066

**Published:** 2013-02-18

**Authors:** Dorothee Wasserberg, Dana A. Uhlenheuer, Pauline Neirynck, Jordi Cabanas-Danés, Jan Hendrik Schenkel, Bart Jan Ravoo, Qi An, Jurriaan Huskens, Lech-Gustav Milroy, Luc Brunsveld, Pascal Jonkheijm

**Affiliations:** 1 Molecular NanoFabrication Group, MESA+ Institute for Nanotechnology, University of Twente, P.O. Box 217, 7500 AE, Enschede, The Netherlands; E-Mails: d.wasserberg@utwente.nl (D.W.); j.cabanasdanes@utwente.nl (J.C.-D.); angeljoy@gmail.com (Q.A.); j.huskens@utwente.nl (J.H.); 2 Laboratory of Chemical Biology, Department of Biomedical Engineering, Eindhoven University of Technology, P.O. Box 513, 5600 MB, Eindhoven, The Netherlands; E-Mails: d.uhlenheuer@gmail.com (D.A.U.); p.neirynck@tue.nl (P.N.); l.milroy@tue.nl (L.-G.M.); 3 Institute of Organic Chemistry, Westfaelische Wilhelms-Universität Muenster, Corrensstrasse 40, 48149 Münster, Germany; E-Mails: j_sche13@uni-muenster.de (J.H.S.); b.j.ravoo@uni-muenster.de (B.J.R.)

**Keywords:** host-guest chemistry, protein immobilization, protein modifications, cyclodextrin, cucurbituril, ferrocene

## Abstract

The supramolecular assembly of proteins on surfaces has been investigated via the site-selective incorporation of a supramolecular moiety on proteins. To this end, fluorescent proteins have been site-selectively labeled with ferrocenes, as supramolecular guest moieties, via SNAP-tag technology. The assembly of guest-functionalized SNAP-fusion proteins on cyclodextrin- and cucurbit [7]uril-coated surfaces yielded stable monolayers. The binding of all ferrocene fusion proteins is specific as determined by surface plasmon resonance. Micropatterns of the fusion proteins, on patterned cyclodextrin and cucurbituril surfaces, have been visualized using fluorescence microscopy. The SNAP-fusion proteins were also immobilized on cyclodextrin vesicles. The supramolecular SNAP-tag labeling of proteins, thus, allows for the assembly of modified proteins via supramolecular host-guest interaction on different surfaces in a controlled manner. These findings extend the toolbox of fabricating supramolecular protein patterns on surfaces taking advantage of the high labeling efficiency of the SNAP-tag with versatile supramolecular moieties.

## 1. Introduction

Assemblies of proteins on arrays, beads, chips, scaffolds and aggregates play an increasingly important role in the development of bioanalytics and biomedicine [1,2]. Protein-modified surfaces are also of interest for the study of cell growth and cell differentiation. The interaction of cells with modified surfaces plays an important role in tissue engineering and the design of medical implanted materials [3]. In addition, the controlled immobilization of enzymes is crucial to optimizing catalytic activities [4]. Supramolecular chemistry is a versatile tool for the assembly of proteins on surfaces, as it in principle allows for a reversible attachment of the proteins to the surface, not accessible via covalent immobilization techniques [5]. Additionally, supramolecular protein immobilization provides entry to control the dynamics and reversibility of the interaction and distribution and density of biological ligands on the surface [5]. Supramolecular protein immobilization using synthetic supramolecular elements, such as adamantine [6] and ferrocene [5,7], attached to the protein and immobilized on the surface of interest, such as cyclodextrins and cucurbiturils, has been reported, and makes use of non-natural recognition elements [8–12]. Most of these approaches rely on multivalent interactions of multiple guest molecules often randomly attached to the protein of interest, making molecular control and uniformity more difficult to achieve. Recently, we reported how expressed protein ligation can be used for the site-selective incorporation of single supramolecular elements into proteins [13–17], including a ferrocene unit and the reversible immobilization of this ferrocene-conjugated protein on supramolecular cucurbituril and cyclodextrin surfaces [5,17]. Site-selective and mono-functional protein conjugation of supramolecular tags offers distinct advantages over random protein labeling and new and versatile methods to incorporate supramolecular elements are therefore urgently needed. A highly interesting approach in this respect is the labeling of proteins using a SNAP-tag, a genetically encoded protein tag developed for the highly efficient labeling of proteins with small molecules featuring an *O*^6^-benzylguanine label [18,19]. In the labeling reaction, the substituted benzyl group of the substrate is covalently attached to a cysteine residue within the guanine binding pocket of the SNAP-tag. This technology has found wide-spread application in different areas of protein and cell biological research [20], including the covalent immobilization of purified fusion proteins on benzylguanine-modified surfaces [21–25]. SNAP-tag technology is a promising candidate for *in vivo* functionalization of proteins expressed within cells or for pull-down assays. Recently, we have adopted this strategy to label fluorescent proteins with adamantanes [26]. Here we want to combine the high chemical specificity and reactivity of the SNAP-tag fusion proteins with the β-cyclodextrin (β-CD)- and cucurbit [7]uril (CB [7])-ferrocene host-guest interaction for rapid and facile entries for site-selective and reversible protein immobilization. To this end, mono- and bivalent supramolecular ferrocene ligands are conjugated to an *O*^6^-benzylguanine derivative, which reacts with the enzyme alkylguanine transferase (SNAP-tag) that is fused to the protein of interest, in this case cyan fluorescent protein (CFP). The fusion proteins are then used for directed assembly on patterned β-CD and CB [7] monolayers as well as on vesicles composed of amphiphilic CDs [27].

## 2. Results and Discussion

### 2.1. Synthesis of Ferrocene Modified Benzylguanine-Conjugates

**Figure 1:**
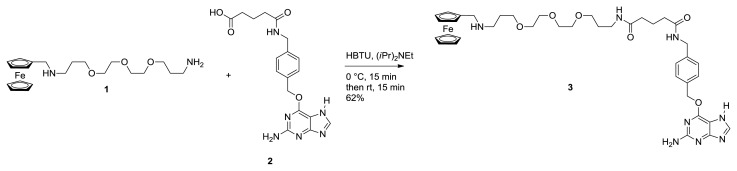
Synthesis of the monovalent ferrocene guest molecule **3**.

**Figure 2:**
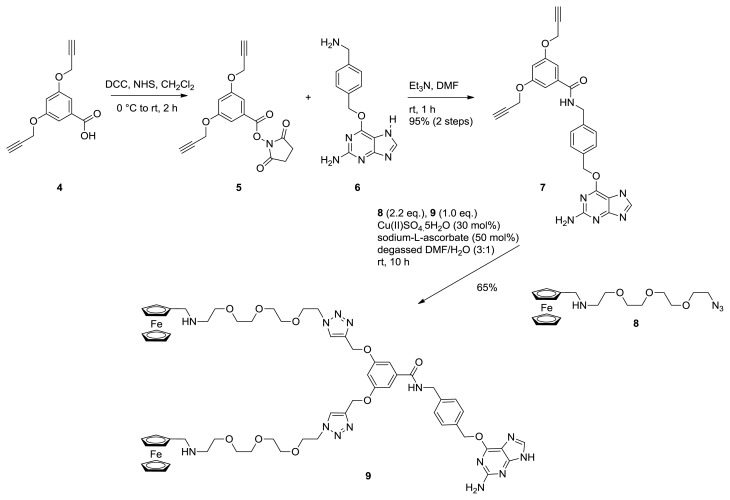
Synthesis of the bivalent ferrocene guest molecule **9**.

To be able to employ the SNAP-tag technology for the modification of proteins with supramolecular moieties, these moieties must be functionalized with *O*^6^-benzylguanine, which later reacts with the enzyme alkylguanine transferase (SNAP-tag) that is fused to the proteins of interest. The monovalent ferrocene guest molecule (**3**) was synthesized using standard amide coupling chemistry (Figure 1) [28]. Thus, the previously reported ferrocene-derived diamine **1** [17] was coupled to the benzylguanine derivative **2** [29] using HBTU in DMF at 0 °C to afford the monovalent guest molecule **3** in a reasonable (62%) yield after purification by silica gel column chromatography [30]. The preactivated acid **2** was added drop-wise to the DMF solution of amine **1** to suppress undesired coupling at the secondary amine. In contrast to the monovalent ferrocene guest molecule, the bivalent variant was prepared using an alternative strategy (Figure 2). First, the known bis alkyne derived benzoic acid **4** [31] was converted to the corresponding *N*-hydroxysuccinimide ester (**5**) using DCC in dichloromethane, which was subsequently reacted with known benzylguanine amine **6** [32] to afford amide **7** in a 95% yield over both steps. Next, two ferrocene molecules were attached using a copper-catalyzed azide-alkyne cycloaddition reaction.[33] Thus, one equiv. of bis alkyne **7** and a slight excess (2,2 equiv.) of azide **8**—readily prepared in one step, starting from commercially available ferrocenealdehyde—were taken up in a freshly degassed 3/1 DMF/H_2_O solvent mixture and treated with 30 mol% CuSO_4_ and 50 mol% sodium ascorbate. After 10 h stirring at room temperature, LC-MS analysis showed conversion of **7** to the target bivalent ferrocene guest molecule **9**. Evaporation of the reaction solvents and purification by silica gel column chromatography afforded pure **9** in a useful 65% yield, which was sufficient for the planned SNAP-tag ligation studies.

### 2.2. Ligation to Proteins

The fluorescent protein that was selected for the oriented immobilization studies was CFP (cyan fluorescent protein). CFP was expressed as a *C*-terminal SNAP-tag fusion protein in *E. coli* (see the Experimental Section). This fluorescent SNAP-fusion protein was labeled with the supramolecular moieties (see Section 2.1) by reacting it with a 5 to 6-fold excess of either of the benzylguanine derivatives [26]. Within two hours at 37 °C the reactions had reached completion as determined by LC/MS (Figures 6 and 7). The excess of the benzylguanine derivatives was easily removed by exchanging the buffer to yield pure mono- and bisferrocene-functionalized proteins, FcSNAPCFP and Fc_2_SNAPCFP. Purity and integrity of all ligated proteins was confirmed by LC/MS. These results demonstrated that selective protein labeling with supramolecular ligands using the SNAP-tag technology is rapid and highly efficient, yielding pure proteins that can be used without the need for any further purification.

### 2.3. Immobilization of Proteins

**Figure 3:**
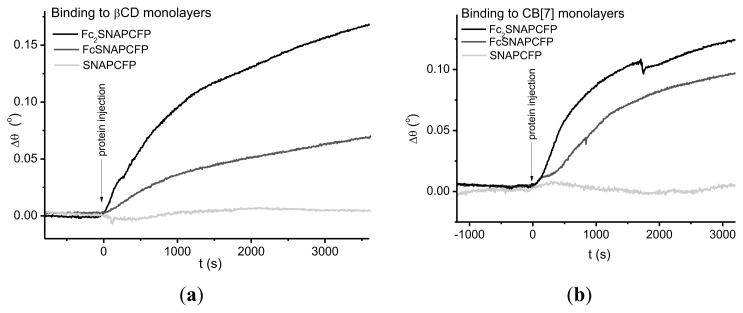
SPR sensorgrams of the immobilization of SNAPCFP (5 μM), FcSNAPCFP (5 μM) and Fc_2_SNAPCFP (5 μM) on (**a**) βCD and (**b**) CB [7] monolayers in running buffer (RB) (PBS with Tween20 (0.005%)).

Surface plasmon resonance (SPR) studies were performed to investigate the interaction between the fluorescent SNAP-fusion protein carrying the supramolecular moieties and β-CD monolayers. To this end, self-assembled monolayers (SAMs) of β-CD on gold were prepared using heptathioether-β-cyclodextrin as described previously [34,35]. The substrates were mounted in a microfluidic cell containing phosphate buffered saline (PBS) solution. First unlabeled (*i.e.*, ferrocene-free) SNAP-tag fusion protein SNAPCFP was used to analyze nonspecific binding to the substrates. To suppress nonspecific adsorption of SNAPCFP (5 μM), PBS buffer with 0.005% *v*/*v* Tween20 was used as running buffer (RB) (Figure 3a). Addition of 5 μM solutions of FcSNAPCFP and Fc_2_SNAPCFP in RB to the microfluidic cell resulted in significant binding to be observed in the SPR sensorgram (Figure 3a). These results show that ferrocene-functionalized SNAP-tag fusion proteins can bind specifically to cyclodextrin monolayers and that the bivalent construct binds stronger to the receptor surfaces as compared to the monovalent analogue. For the generation of CB [7] monolayers, we applied the recently reported method of the spontaneous adsorbtion of CB [7] directly onto a gold surface [17,36]. Similar specific assembly of the ferrocene-functionalized SNAP-CFPs was observed when assembling these proteins onto such monolayers of CB [7] (Figure 3b). Presumably on this type of CB [7]-surfaces optimal binding configuration is not possible as the CB is attached to the surface on one side affecting the structure of the complex and thus its binding strength. In the case of CD, this restriction should be of minor importance as the CD is attached to the surface using long linkers.

**Figure 4:**
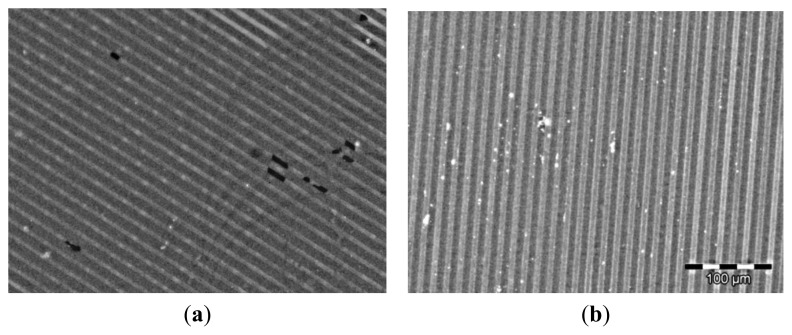
Fluorescent microscopy images of FcSNAPCFP on (**a**) patterned CD and (**b**) patterned CB [7]-surfaces. Scale bare applies to both images and bright areas correspond to the blue fluorescence of the CFP whereas dark areas correspond to non-fluorescent polyethyleneglycol areas.

The applicability of the ferrocene-labeled fluorescent SNAP fusion proteins for supramolecular assembly on surfaces was demonstrated via the formation of homogeneous patterns of the Fc-functionalized fusion proteins on β-CD and CB [7] patterned surfaces via supramolecular recognition. Using standard UV-photolithography procedures line patterns of photoresist on glass were fabricated (see supporting information). β-CD was attached to the glass parts of the pattern and after removal of the residual photoresist, the other parts of the pattern were filled with a biochemically inert poly(ethylene glycol) silane. Bovine serum albumin blocked surfaces were immersed for 90 min in a 2 μM solution of FcSNAPCFP. Homogeneous and intensely fluorescent patterns were observed (Figure 4a), whereas no binding of SNAPCFP, which lacks the ferrocene moiety, was observed on the surface (data not shown). Similarly, patterns of Fc_2_SNAPCFP were achieved (data not shown). For the immobilization of the proteins on patterned CB [7] surfaces, 15 μm wide gold lanes were fabricated, separated by 5 μm of bare glass, which was subsequently modified with a poly(ethylene glycol) silane. After immersing these substrates in a saturated solution of CB [7], CB [7] was adsorbed on gold. Subsequently, these CB [7] monolayer patterns were immersed for 90 min in a 2 μM solution of FcSNAPCFP. The recorded images show clear fluorescent lines matching the pattern of the gold (Figure 4b). These results show that the ferrocene-functionalized fluorescent SNAP-tag fusion proteins can be used to fabricate protein patterns through specific interaction with different supramolecular hosts in a facile and rapid manner, via simple incubation.

### 2.4. Immobilization on Vesicles

**Figure 5:**
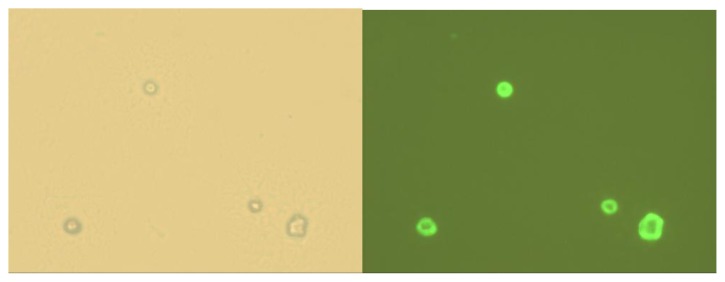
Bright field (**left**) and fluorescence (**right**) microscopy images of cyclodextrin vesicles (100 μM) incubated with Fc_2_SNAPCFP (10 μM). Field of view 100 × 100 μm.

As a next step, the interaction of Fc_2_SNAPCFP with multilamelar cyclodextrin vesicles, formed by amphiphilic β-CD in buffered solution, was studied. The multilamellar CD vesicles ([CD] = 100 μM; for preparation see the Experimental Section) were incubated with Fc_2_SNAPCFP and studied using fluorescence microscopy (Figure 5). Whereas bare vesicles and vesicles incubated with SNAPCFP lacking the ferrocene anchors were only visible in transmission mode, vesicles that were incubated with Fc_2_SNAPCFP were also fluorescently emissive. The vesicles incubated with SNAPCFP only showed background fluorescence of the protein in solution. The vesicles incubated with Fc_2_SNAPCFP showed a clear signal enhancement at the vesicles, showing the preferential recruitment of the bisferrocene-functionalized protein to the surface of the vesicles. These results show that the Fc_2_SNAPCFP was bound specifically to the vesicles via the interaction of ferrocene and cyclodextrin after simple mixing of the vesicles with the proteins.

## 3. Experimental Section

### 3.1. Synthesis and Characterization

#### 3.1.1. Monovalent Ferrocene Guest Molecule **3**

Benzylguanine derived carboxylic acid **2** [29] (50 mg, 0.13 mmol) was dissolved in 2 mL dry DMF and incubated with 49 mg (0.13 mmol) HBTU and 22.5 μL (0.13 mmol) DIPEA for 15 min at room temperature. The mixture was slowly added to a solution of 57 mg (0.13 mmol) of ferrocene derived diamine **1** [17] in 4 mL of dry DMF at 0 °C. The mixture was stirred for an additional 15 min. Then 10 μL of TFA were added to the mixture and the solvent was removed at 40 °C under reduced pressure. The crude product was adsorbed on silica and purified via column chromatography using 10% MeOH/CH_2_Cl_2_ and 10% MeOH/CH_2_Cl_2_ with 1% NH_3_ (28% in H_2_O) to obtain 63 mg (0.08 mmol, 62%) of the pure product, **3** [30] as a orange-yellow film. ^1^H NMR (400 MHz, CD_3_OD) δ = 7.82 (s, 1H, H8′), 7.37 (m, 4H, Ar), 5.52 (s, 2H, BzO), 4.35 (s, 2H, BzNH), 4.21 (m, 2H, Cp), 4.11 (s, 7H, Cp), 3.64–3.41 (m, 14H, -CH_2_O-, FcCH_2_), 3.23 (t, *J* = 6.8, 2H), 2.68 (t, *J* = 7.0, 2H) 2.22 (m, 4H, CH_2_CO(BG)), 1.97–1.83 (m, 4H, COC*H*_2_-CH_2_-C*H*_2_CO), 1.73 (m, 2H, CH_2_C*H*_2_CH_2_); ^13^C-NMR (100 MHz, CD_3_OD) δ 175.19, 175.11, 161.58, 161.32, 157.20, 140.31, 140.03, 136.92, 129.66, 128.67, 113.46, 85.45, 71.49, 71.47, 71.20, 70.76, 70.16, 69.87, 69.53, 69.16, 68.59, 49.54, 47.51, 43.83, 37.82, 36.30, 36.20, 30.39, 29.85, 23.26. MALDI-TOF: C_39_H_52_FeN_8_O_6_ Calcd. M = 784.34, Found: [M + H]^+^ 784.41, [M + Na]^+^ 807.41.

#### 3.1.2. NHS Ester **5**

*N*-hydroxysuccinimide, NHS (600 mg, 5.21 mmol, 1.2 equiv.) was added to a solution of acid **4** (1.0 g, 4.34 mmol) [31] in dry dichloromethane (80 mL). The mixture was stirred at room temperature under a positive argon pressure for 15 min and then cooled to 0 °C. *N*,*N*′-Dicyclohexylcarbodiimide (DCC) was then added as a single portion (1.12 g, 5.43 mmol, 1.25 equiv.) and the resulting mixture allowed to warm to room temperature while stirring overnight. After removal of the white precipitate by filtration (washing with dichloromethane, to give a total solvent volume of 200 mL), the organic solvents were washed with sat. aq. NaHCO_3_ (50 mL), brine (50 mL) and then dried over MgSO_4_ and evaporated under reduced pressure to constant mass to yield the crude material. This was purified by silica gel column chromatography (cyclohexane/ethyl acetate) to afford the target compound **5** as a white solid (1.35 g, 4.12 mmol, 97%).^1^H NMR (400 MHz, *d^6^*-DMSO): δ = 7.29 (d, *J* = 4 Hz, 2H), 7.08 (t, *J* = 4 Hz, 1H), 4.94 (d, *J* = 4 Hz, 4H), 3.63 (t, *J* = 4 Hz, 2H), 2.90 (s, 4H) ppm; ^13^C NMR (100 MHz, *d^6^*-DMSO): δ = 170.20, 161.32, 158.62, 126.26, 109.65, 108.90, 78.96, 78.53, 56.09, 25.54 ppm.

#### 3.1.3. Bis Alkyne Benzylguanine **7**

NHS ester **5** (73 mg, 0.222 mmol, 1.2 equiv.) was added to a 25 mL round-bottomed flask fitted with a magnetic stir bar and then dissolved in 2 mL dry DMF. Benzylguanine derivative **6** (50 mg, 0.185 mmol) was added, followed by dry Et_3_N (130 μL, 0.925 mmol, 5.0 equiv.) and the reaction stirred at room temperature under a positive argon pressure. Analysis of the reaction by TLC (MeOH/CH_2_Cl_2_) after 1 h revealed completed conversion of **2**. Therefore, the reaction was diluted with 10 mL EtOAc and 2 mL H_2_O, the organic and aqueous layers separated, and the aqueous phase extracted with 3 × 5 mL EtOAc. The combined organic layers were washed with brine, dried over MgSO_4_ and filtered to afford the crude material, which was purified by silica gel column chromatography to yield the bis alkyne benzylguanine intermediate **7** as a white powder (87 mg, 0.180 mmol, 97%). ^1^H NMR (400 MHz, *d4*-MeOH): δ = 7.83 (broad s, 1H), 7.51 (d, *J* = 8 Hz, 2H), 7.37 (d, *J* = 8 Hz, 2H), 7.12 (d, *J* = 4 Hz, 2H), 6.80 (t, *J* = 4 Hz, 1H), 5.54 (s, 1H) 4.77 (d, *J* = 4 Hz, 4H), 4.57 (s, 2H), 2.97 (t, *J* = 4 Hz, 2H) ppm; ^13^C NMR (400 MHz, *d4*-MeOH): δ = 165.41, 159.82, 159.61, 158.16, 155.17, 139.32, 137.76, 136.35, 135.29, 128.55, 127.30, 106.72, 105.03, 78.94, 78.49, 65.51, 55.79, 42.53 ppm. LC/MS *R*_t_ = 5.25 min, C_26_H_22_N_6_O_4_ Calcd. [M + H]^+^ = 483.17, found = 483.10.

#### 3.1.4. Bis Ferrocene Benzylguanine **9**

Bis alkyne benzylguanine derivative **7** (10 mg, 0.0207 mmol) and ferrocenyl azide **8** (22 mg, 0.0518 mmol, 2.5 equiv.) were weighed into a 10 mL glass round-bottomed flask fitted with a magnetic stir bar, and dissolved in 0.5 mL of a 3/1 solvent mixture of thoroughly degassed DMF/H_2_O. Sodium ascorbate (2.1 mg, 50 mol%) and CuSO_4_·*x*H_2_O (1.0 mg, 30 mol%) were added and the reaction stirred at room temperature under argon. After 10 h, LC-MS analysis of the reaction revealed complete consumption of the bis alkyne **7** starting material. The solvents were evaporated and the reaction mixture purified by silica gel column chromatography (7% MeOH in CH_2_Cl_2_ with 1% NH_4_OH) to yield the target compound **9** as a yellow orange film (18 mg, 0.0135 mmol, 65%). ^1^H NMR (400 MHz, *d4*-MeOH): δ = 8.08 (s, 2H), 7.82 (s, 1H), 7.48 (d, *J* = 8 Hz, 2H), 7.36 (d, *J* = 8 Hz, 2H), 7.17 (d, *J* = 4 Hz, 2H), 6.85 (t, *J* = 4 Hz, 1H), 5.52 (s) 5.17 (s), 4.57 (s), 4.53 (app. t, *J* = 8 Hz, 4H), 4.16 (t, *J* = 4 Hz, 4H), 4.09 (s), 4.07 (t, *J* = 4 Hz), 3.82 (app. t, *J* = 8 Hz), 3.58–3.48 (m), 3.45 (s), 2.71 (app. t, *J* = 4 Hz) ppm; ^13^C NMR (100 MHz, *d^4^*-MeOH): δ = 167.74, 160.20, 159.90, 159.54, 155.75, 142.96, 138.79, 136.40, 135.52, 128.30, 127.32, 124.85, 111.98, 106.49, 105.05, 85.05, 70.05, 69.99, 69.97, 69.90, 68.97, 68.84, 68.37, 68.09, 67.61, 67.20, 61.31, 53.40, 50.05, 47.87, 42.95, ppm. LC *R*_t_ = 4.72 min (single peak). MALDI-TOF C_64_H_79_Fe_2_N_14_O_10_ Calcd. [M + H]^+^ = 1315.47, found = 1315.25.

### 3.2. Plasmid Construction, Protein Expression and Purification and Ligation

The SNAP-CFP construct is based on one gene construct and expressed as such. The SNAP tag is thus not connected to one of the 8 cysteine residues of the CFP via a chemical reaction, as the reviewer thinks. As a result, there is full molecular control over the constitution of the protein construct we have used for our studies. To construct the plasmids, pHT404 (-HisSNAPCFPStrep), the fragment encoding CFP was amplified by PCR using a pair of primers, ON007, 5′-ggcggatccgtgagcaagggcgaggagct-3′ (underlined, BamHI site) and ON008, 5′-ggcgagctccttgtacagctcgtccatgccg-3′ (underlined, SacI site) with pHT559 as template. The BamHI/SacI-digested product was ligated into pHT402. The plasmid pHT402 (HisSNAPStrep) was constructed by introducing a DNA fragment encoding for the SNAP-tag using a pair of primers, ON005, 5′-ggcggatccggcgagctcgacaaagactgcgaaatgaagcg-3′ (underlined, BamHI site) and ON006, 5′-ggcggaattcacccagcccaggcttgcc-3′ (underlined, EcoRI site) with pSEMS1-26m (Covalys) as a template into pHT401 at BamHI and EcoRI restriction sites. Plasmid pHT404 (HisSNAPCFPStrep) was transformed into *E. coli* BL21 using heat shock for 60 s at 42 °C. Transformants were selected on ampicillin (125 mg/L) agar plates. 10 mL of LB medium containing 100 mg/L ampicillin was inoculated with a single colony and the culture was grown overnight at 37 °C. This preculture was used to seed 2 L of fresh LB medium containing antibiotics, and the culture was incubated at 37 °C until the absorbance at 600 nm (OD600) reached 0.7–0.9. After cooling down to 15 °C, IPTG was added to a final concentration of 0.5 mM, and overnight (or 6 h) induction was performed at 15 °C. Cells were harvested by centrifugation (4500 rpm, rotor SLA-3000, 20 min, 4 °C). The pellet was frozen at −80 °C. After thawing it was resuspended in BugBuster Reagent (Novagen) according to the supplier’s instructions. Benzonase Nuclease was added to the suspension which was then incubated on a shaker for 20 min at room temperature. The lysate was cleared by centrifugation (20,000 rpm, rotor SA300, 30 min, 4 °C). Columns (EconoPac, Biorad, 20 mL) containing 10 mL of HisBind resin (Novagen) were equilibrated according to the supplier’s instructions. The supernatant was applied to the columns at room temperature. The beads were washed with 10–15 column volumes of washing buffer (0.5 M NaCl, 20 mM Tris HCl, 30 mM imidazole, pH 8). The protein was eluted using a buffer containing 0.5 M imidazole but otherwise identical to the washing buffer. The pooled fractions were concentrated and the buffer exchanged three times to storage buffer (25 mM sodium phosphate, 50 mM NaCl, pH 7.5) and the protein concentrated to 10 mg/mL. The protein was aliquoted and snapfrozen using liquid nitrogen and stored at −80 °C. Yields were typically about 20 mg protein per liter culture medium. Proteins were analyzed by LC/MS: Strep-ECFPSNAP-His, C_2217_H_3412_N_596_O_649_S_11_ Calcd. MW = 49151, found 49156; and SDS-PAGE.

### 3.3. Protein Ligation

**Figure 6:**
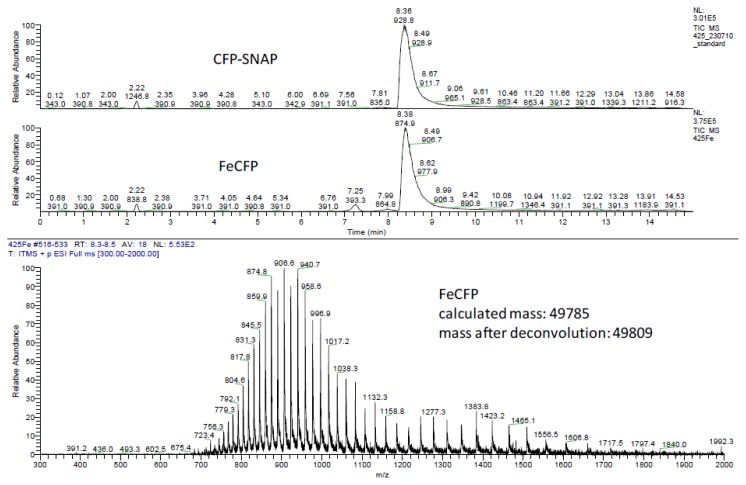
LC-MS of FcSNAPCFP.

SNAPCFP + Ferrocene-benzylguanine **3**: Ferrocene-benzylguanine **3** (5 equiv, in a 1.5 mM solution in MeOH) was ligated to SNAPCFP at a concentration of 40 μM in phosphate buffer (25 mM Na phosphate, 50 mM NaCl), for 2 h at 37 °C. To remove the small molecules, the buffer was exchanged several times using Amicon filters with a MWCO of 10,000 Da. The resulting FcSNAPCFP was characterized using LC-MS (Figure 6) *R*_t_ 8.4 min, C_2251_H_3459_FeN_599_O_654_S_11_ Calcd. MW = 49785, found 49809.

**Figure 7:**
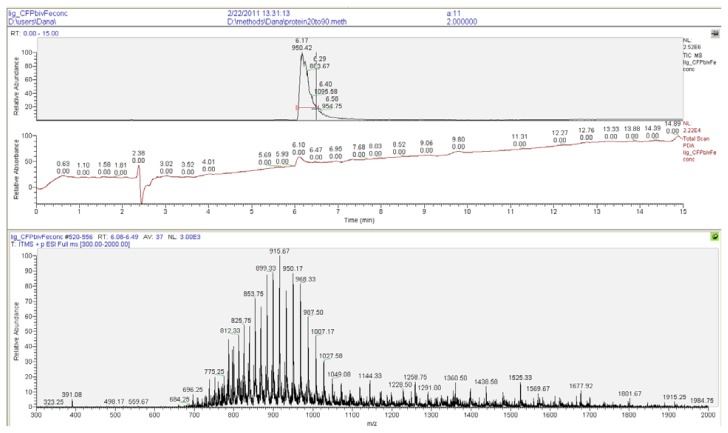
LC-MS of Fc_2_SNAPCFP.

SNAPCFP + Bisferrocene-benzylguanine **9**: Bisferrocene-benzylguanine **9** (6.6 equiv., 15.2 mM, 50 μL) was ligated to SNAPCFP (230 μM, 500 μL) in 2.5 mL ligation buffer at 37 °C for 2 h. To remove the small molecules, the buffer was exchanged several times using Amicon filters with a MWCO of 10,000 Da. The resulting Fc_2_SNAPCFP was characterized using LC-MS (Figure 7) *R*_t_ 6.2 min, C_2276_H_3486_N_605_O_658_S_11_Fe_2_ Calcd. MW = 50316, found 50311.

### 3.4. Surface Plasmon Resonance

β-cyclodextrin surfaces were prepared according to published procedures [34,35]. The CB [7] SAM was prepared as described previously [17]. Prior to further use, they were washed with Millipore water and ethanol and dried over a stream of nitrogen. Surface plasmon resonance (SPR) measurements were performed in an SPR setup in Kretschmann configuration. Glass substrates covered with a 50 nm gold layer and a CD or CB monolayer were attached to a 70 μL volume microfluidic cell. SPR experiments were performed at a continuous flow of 20 μL/min. The resonance angle was determined by continuously scanning through the surface plasmon resonance dip.

### 3.5. Preparation of Patterned Host Surfaces by UV-Lithography

A piranha cleaned 4 inch borofloat wafer was spin-coated with hexamethyldisilane (HMDS) for 30 s at 4000 rpm. On top, the photoresist Olin 907-17 (FujiFilm) was spin-coated for 30 s at 4000 rpm. To remove residual solvents, the wafer was prebaked at 95 °C for 90 s. Subsequently the Olin resist coated wafer was illuminated through a photomask using an EVG 620 mask aligner with UV-light for 4 s (12 mW/cm^2^ Hg-lamp). After exposure, the wafer was baked for 10 min at 120 °C and then developed during 60 s in OPD-4262. The wafers were rinsed with copious amounts of water, dried and stored in a nitrogen box until further use. For the formation of the amine-terminated monolayer on the bare surface of the substrate, the complete wafer was cut into substrates of *ca.* 1 cm^2^ size. Silanization was carried out by overnight chemical vapor deposition of (trimethoxysilyl)propyl-ethylenediamine (TPEDA) in vacuo. To remove the excess of silane, the samples were rinsed thoroughly with ethanol. The amine-terminated monolayer was reacted with phenyl diisothiocyanate (ITC). The samples were immersed in a 0.04 M solution of ITC in ethanol for 3 h at 50 °C under argon. After the reaction, the samples were rinsed with ethanol and dried in a stream of nitrogen. The ITC-terminated monolayer was then reacted with β-cyclodextrin-heptaamine [37]. To this end, the samples were incubated in a 1 mM solution of β-cyclodextrin-heptaamine in water at 40 °C for 4 h under argon. Samples were washed afterwards with water and dried in a stream of nitrogen. To remove the Olin resist from the remaining lines, the samples were immersed in acetone and sonicated for 2.5 h. Samples were rinsed with acetone and dried in a stream of nitrogen. To check if the resist had been removed completely, optical microscopy images were taken. To form a PEG-terminated monolayer on the now bare (second) part of the pattern, the samples were incubated in a solution of 100 μL PEG-(trimethoxy)silane (AB111226 from ABCR) in 60 mL dry toluene overnight under argon at room temperature. Samples were then washed with toluene and stored in a nitrogen box until use for protein immobilization.

A bilayer lift-off recipe was used for fabricating gold lanes on borofloat glass wafers. First LOR 5A (MicroChem) was spincoated, after which normal lithography was done on these wafers with Olin OiR 907-17 photoresist (FujiFilm), creating a bilayer resist stack. Gold patterns were created by exposing the photoresist through a patterned photomask and developing Olin in OPD 4262. The developing step washed away the exposed photoresist and also etched through the LOR 5A layer, creating an undercut in the second resist. Then 3 nm Ti and 17 nm Au were deposited via e-beam evaporation (BAK 600, Balzers). The bilayer resist was then removed and thus used as a sacrificial layer, leaving gold lanes on borofloat glass. Then, the remaining areas of bare glass on the substrate was modified using poly(ethylene glycol) silane by incubation with a solution of 2% AB111226 (% *v*/*v*) in dry toluene for 2 h at 60 °C. After washing with toluene and drying, the substrate was baked at 120 °C for 2 h and stored under nitrogen until protein immobilization.

### 3.6. Protein Immobilization on Patterned Surfaces

The substrates were first incubated in a humidity chamber with a solution of 1 mg/mL bovine serum albumine (BSA) in PBS with 0.005% Tween20 (PBST) for 30 min. The solution was removed with a pipette from the surface and the samples were rinsed three times with PBST and washed two times for 10 min with PBST by incubation on an orbital shaker at 80 rpm. Then, the substrates were incubated in a humidity chamber with a 2 μM protein solution (SNAPCFP or FcSNAPCFP or Fc_2_SNAPCFP) for 90 min. The solution was removed with a pipette and the samples were washed on an orbital shaker in PBST for at least 1 h. The samples were rinsed with water and dried in a stream of nitrogen. The samples were then imaged using an Olympus IX70 inverted fluorescence microscope with a Hg-lamp as light source using appropriate filters (λ_ex_ = 425–450 nm and λ_em_ = 460–500 nm).

### 3.7. Cyclodextrin Vesicles

CD-vesicles were prepared as described in the literature [26]. Fluorescence microscopy was done with a CKX41 inverted microscope from Olympus equipped with a Hg-lamp (U-RFL-T from Olympus). Pictures were taken with a DX 20 L-FW camera (Kappa opto-electronics GmbH, Gleichen, Germany). Vesicles were mixed with the protein solutions as indicated above and studied using a fluorescence microscope.

## 4. Conclusions

Fluorescent proteins were effectively and easily modified with the supramolecular guest molecule ferrocene in a site selective manner employing SNAP-tag technology. The mono- and bisferrocene SNAP-tag fluorescent fusion proteins were successfully immobilized on β-CD and CB [7] monolayers. Using patterned β-CD and CB [7] surfaces it could be shown that the ferrocene-functionalized proteins specifically bind via their supramolecular elements to the surface, generating the corresponding uniform fluorescent protein patterns. Additionally it could be shown that the modified proteins bind to vesicles formed by amphiphilic CDs. The combination of supramolecular elements with the SNAP-tag technology for selective protein labeling is, thus, an efficient approach for labeling proteins with designed supramolecular elements. This leads to valuable materials for effective protein immobilization onto a variety of surfaces.
